# Toward regionally equitable precision medicine: microbiota‑associated mechanisms and emerging personalized strategies in multiple sclerosis

**DOI:** 10.1186/s12967-026-08830-7

**Published:** 2026-10-08

**Authors:** Ameera Saeed Alshinnawy, Elham AbdelBadiea Rashwan, Mahmoud Saad Swelam, Mohamed R. Mohamed, Ahmed A. Sayed

**Affiliations:** 1 Department of Biochemistry, Faculty of Science, Ain Shams University, Cairo, 11566 Egypt; 2 Children’s Cancer Hospital, Cairo, 57357 Egypt; 3 Department of Neurology, Faculty of Medicine, Ain Shams University, Cairo, Egypt

**Keywords:** Multiple sclerosis, Gut microbiota, Regional disparities, Sequencing, Immune modulation, Precision medicine

## Abstract

Multiple sclerosis (MS) is a chronic immune-mediated disorder of the central nervous system in which dysregulated immune signalling and barrier dysfunction play central pathogenic roles. Increasing evidence implicates the gut microbiota as a key modulator of host immune homeostasis; however, current understanding is largely derived from Western cohorts and remains fragmented at the mechanistic level. This review synthesizes global evidence alongside emerging data from underrepresented regions, including Egypt, Iran, and India, to delineate molecular pathways linking MS-associated dysbiosis to immune dysregulation. Across studies, dysbiosis is consistently associated with impaired short-chain fatty acid–mediated immunoregulation, skewing toward proinflammatory Th1/Th17 responses, and disruption of intestinal and blood–brain barrier integrity, with region-dependent functional signatures involving oxidative stress and neurotransmitter and tryptophan metabolism. Several regional signatures reported here derive from a single pilot-scale cohort and should be regarded as hypothesis-generating pending independent validation, and current microbiota-targeted interventions remain investigational rather than established disease-modifying therapies for MS. We critically examine microbiota-derived signals, including short-chain fatty acids, lipopolysaccharide, and polysaccharide A, that modulate dendritic cell programming, regulatory T-cell differentiation, and neuroinflammatory cascades. We further evaluate microbiome-targeted therapeutic strategies and discuss how sex, regional context, and sequencing methodology influence functional interpretation and therapeutic responsiveness. Finally, we propose a roadmap for regionally equitable precision medicine, advocating for standardized multi-omics and culturally inclusive longitudinal designs to move beyond empiric interventions toward personalized, strain-defined biotherapeutics tailored to the unique microbial and environmental landscapes of MS patients worldwide.

**Graphical Abstract:**

**Figure:**
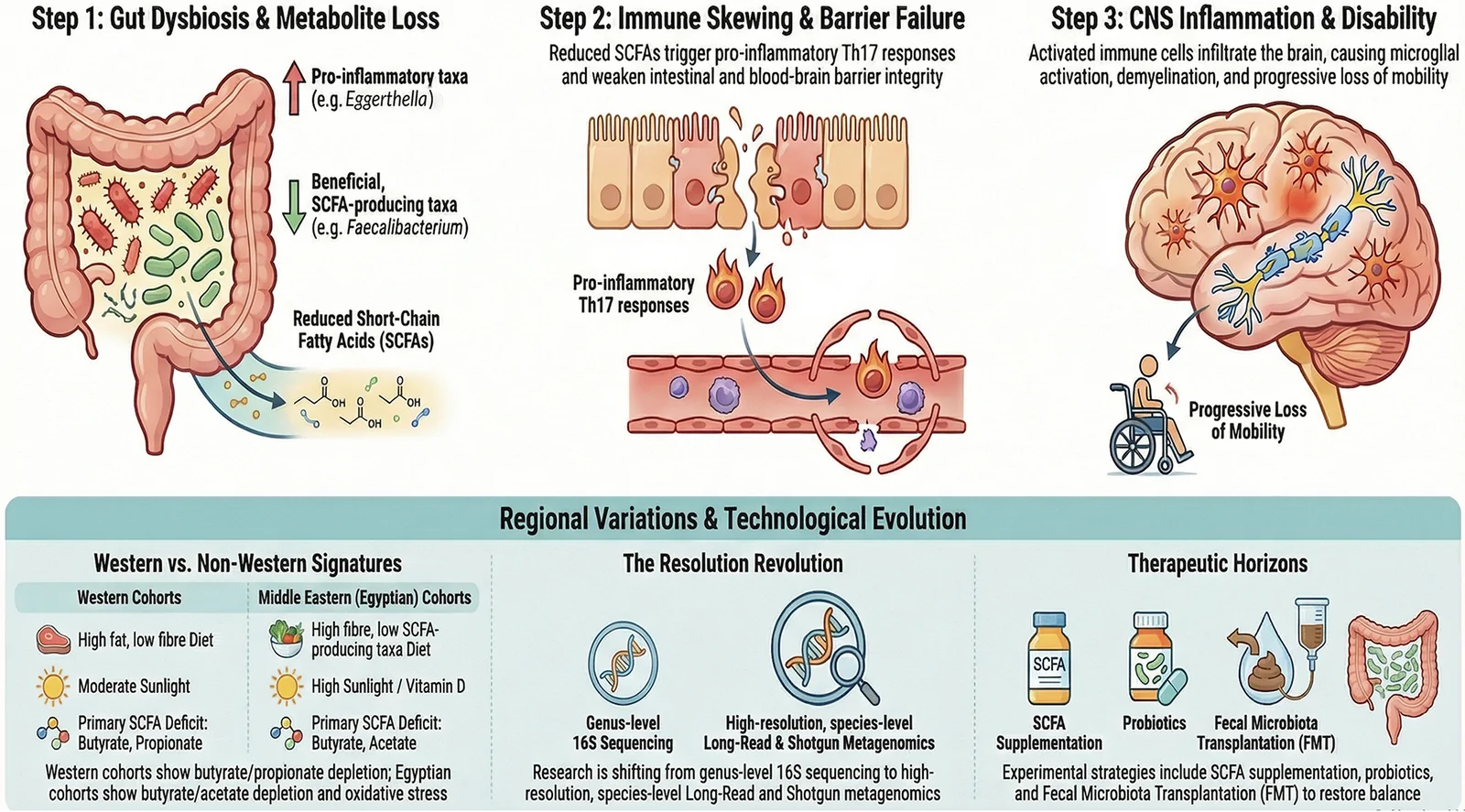

## Introduction

Multiple sclerosis (MS) is a chronic immune-mediated disease of the central nervous system (CNS) affecting over 2.8 million people worldwide, with a higher burden among young adults and women [1, 2]. Although MS has historically been reported more frequently in Western populations, prevalence estimates have increased across world regions in recent Atlas based analyses, including the Middle East and North Africa (MENA). According to the MS International Federation (MSIF) Atlas of MS (3rd edition; country-level data collected via surveys of national representatives between September 2019 and March 2020), an estimated 59,671 people are living with MS in Egypt (approximately 1 in 1,500, ≈ 0.067%) [3, 4]. This increasing incidence highlights the urgency of region-specific investigations. Importantly, MS is also one of the leading causes of non-traumatic disability in young adults, with progression commonly tracked using the Expanded Disability Status Scale (EDSS). Understanding how gut microbiota alterations may accelerate disability progression adds crucial clinical context to microbiome studies.

MS is now recognized as a multifactorial disease shaped by genetic susceptibility, environmental exposures, and immune dysregulation. Among these factors, the gut–brain axis has emerged as a central pathway of interest. Through the production of microbial metabolites and dynamic host–microbe interactions, the gut microbiota can modulate immune function, maintain intestinal barrier integrity, and influence neuroinflammatory pathways, thereby positioning it as a potential contributor to the pathogenesis of Multiple Sclerosis [5, 6]. Dysbiosis, including loss of short-chain fatty acids (SCFAs) producing taxa and expansion of pro-inflammatory microbes, has been consistently associated with MS [7, 8].

Most microbiome studies in MS have focused on Western cohorts, yet emerging evidence suggests that regional factors including diet, lifestyle, and genetic diversity shape distinct microbial configurations in non-Western populations. Our recent Egyptian studies using long-read sequencing revealed enrichment of pro-inflammatory taxa and depletion of SCFA-producing taxa (*Faecalibacterium duncaniae*, *Ruminococcus bromii*) [9]. It is important to emphasize that this Nanopore-based dataset derives from a single, pilot-scale Egyptian cohort. Several of the region-specific conclusions drawn in this review rest heavily on this one study, and these findings should be regarded as preliminary and hypothesis-generating rather than confirmatory until independently replicated in larger, multi-center Egyptian and MENA cohorts. These region-specific findings underscore the importance of moving beyond Western-centric models toward culturally and geographically tailored approaches to MS research. Accordingly, this review integrates global evidence with emerging data from Egypt, Iran, and India to illustrate how gut microbiota alterations contribute to MS pathophysiology. We further assess sequencing technologies and microbiota-targeted interventions, highlighting regional differences as opportunities for biomarker discovery, therapeutic development, and the advancement of equitable, personalized strategies in MS. Where country level MS microbiome datasets remain limited (particularly in Iran and India), we also draw on broader MENA/South Asian evidence and clearly distinguish between country specific findings and regional patterns.

### Search strategy

This narrative review is based on a structured search of PubMed/MEDLINE, Scopus, and Web of Science from database inception to March 2026, using combinations of the terms “multiple sclerosis,” “gut microbiota/microbiome,” “dysbiosis,” “short-chain fatty acids,” “gut–brain axis,” and region terms (“Egypt,” “Iran,” “India,” “MENA,” “South Asia”). We prioritized primary human studies and mechanistic work, supplemented by key preclinical and meta-analytic sources; non-English records and conference abstracts without full data were excluded.

## Gut microbiota as a regulator of autoimmune pathogenesis

The gut microbiota is increasingly recognized as a central regulator of systemic immune homeostasis through microbe host interactions and metabolites (e.g., SCFAs, tryptophan derivatives, and bile acid derived ligands) that shape regulatory T-cell (Treg) differentiation and inflammatory cytokine balance [5, 10]. Dysbiosis has been associated with increased intestinal permeability and aberrant immune activation across autoimmune conditions, providing a general framework for microbe driven immune dysregulation [11]. Similarly, in MS, accumulating human and experimental evidence links dysbiosis to reduced immunoregulatory signaling (including SCFA-associated pathways), skewing toward proinflammatory Th1/Th17 responses, and barrier dysfunction that may facilitate systemic immune activation with downstream neuroinflammatory effects [12]. MS cohorts consistently report depletion of beneficial taxa (e.g., *Faecalibacterium* and *Prevotella*) alongside enrichment of proinflammatory taxa (e.g., *Enterococcus* and *Eggerthella*), with functional implications for immune regulation and neuroinflammation [7, 8, 13]. Collectively, current evidence suggests that MS-associated dysbiosis is linked to reduced SCFA-mediated immunoregulation (including diminished Treg tone), proinflammatory Th1/Th17 polarization, and intestinal/ blood-brain barrier (BBB) disruption, supporting a gut–immune–brain axis that may contribute to MS susceptibility and disease activity.

## MS pathophysiology and the gut–brain axis

MS is characterized pathologically by chronic inflammation, demyelination, gliosis, and axonal degeneration within the CNS. These processes underpin the clinical forms of the disease, including relapsing-remitting MS (RRMS), secondary progressive MS (SPMS), and primary progressive MS (PPMS) [14]. At the mechanistic level, disease activity reflects an interplay of autoreactive T and B lymphocytes, microglial activation, and disruption of neuroprotective pathways. Increasing evidence highlights the gut–brain axis (GBA) as a major contributor to these immune processes, moving beyond CNS focused models of pathogenesis.

The gut microbiota exerts profound effects on host immunity, shaping both innate and adaptive responses. In MS, dysbiosis is increasingly recognized as a key environmental factor that disrupts immune homeostasis, enhances neuroinflammation, and weakens barrier integrity [15]. These pathways can be activated through the GBA, a bidirectional communication system involving neural, immune, and endocrine signaling [5], in which dysbiosis can weaken the intestinal barrier and increase permeability (“leaky gut”). Barrier disruption associated with dysbiosis may increase systemic exposure to microbial components and metabolites (e.g., lipopolysaccharide. (LPS) and other microbe-associated molecular patterns, as well as SCFAs), which can promote peripheral immune activation and particularly when BBB integrity is compromised may facilitate neuroinflammatory responses through microglial activation and immune cell trafficking into the CNS [6, 13].

The transition from homeostasis to pathogenesis is driven by a metabolic-structural breakdown. MS-associated dysbiosis is characterized by the enrichment of pro-inflammatory taxa such as *Akkermansia muciniphila*, *Enterococcus faecium*, and *Eggerthella lenta*, alongside a depletion of anti-inflammatory commensals including *Faecalibacterium prausnitzii*, *Prevotella copri*, and *Bacteroides fragilis* [7, 8].

The role of Akkermansia in MS remains contested and illustrates how the same taxon can be assigned opposing functional labels depending on strain, host compartment, and experimental model. In murine EAE, Akkermansia strains from MS patients reduced IL-17⁺ γδ T cells and ameliorated disease [8], whereas a mechanistically distinct pathway showed that *A. muciniphila*-driven mucin degradation liberates mucin-derived sugars that cross-feed the tryptophan-utilizing genus *Alistipes*, generating aryl hydrocarbon receptor (AhR)-agonist indole metabolites that drive AhR-dependent Th17 differentiation, elevate IL-17 A production, and exacerbate EAE severity [16]. Colonization outcome is not fixed even for a single strain: the same inoculum exacerbated EAE and depleted SCFA-producing Clostridia in a Clostridia-rich background microbiome but had no effect in a Clostridia-low background [17], and live (but not heat-inactivated) A. muciniphila selectively protected against colitis via a retinoic acid–IL-22 axis [18]. Notably, all of this evidence derives from murine EAE or colitis models rather than human MS cohorts. Taken together, Akkermansia’s net effect on autoimmune disease depends on strain identity, the resident microbial community, and viability; claims about its role in MS should specify the strain, background microbiome, and model system invoked rather than treat the organism as uniformly pro- or anti-inflammatory.

Additionally, the inclusion of *Bacteroides fragilis* among anti-inflammatory commensals refers specifically to its capsular polysaccharide A (PSA), which promotes FOXP3⁺ regulatory T-cell differentiation and IL-10 production [19, 20] and suppresses CNS demyelination in EAE [21]; we acknowledge that *B. fragilis* also comprises enterotoxigenic strains with opposing pathobiont properties [22], so this attribution is strain-defined rather than species-wide.

**Fig. 1:**
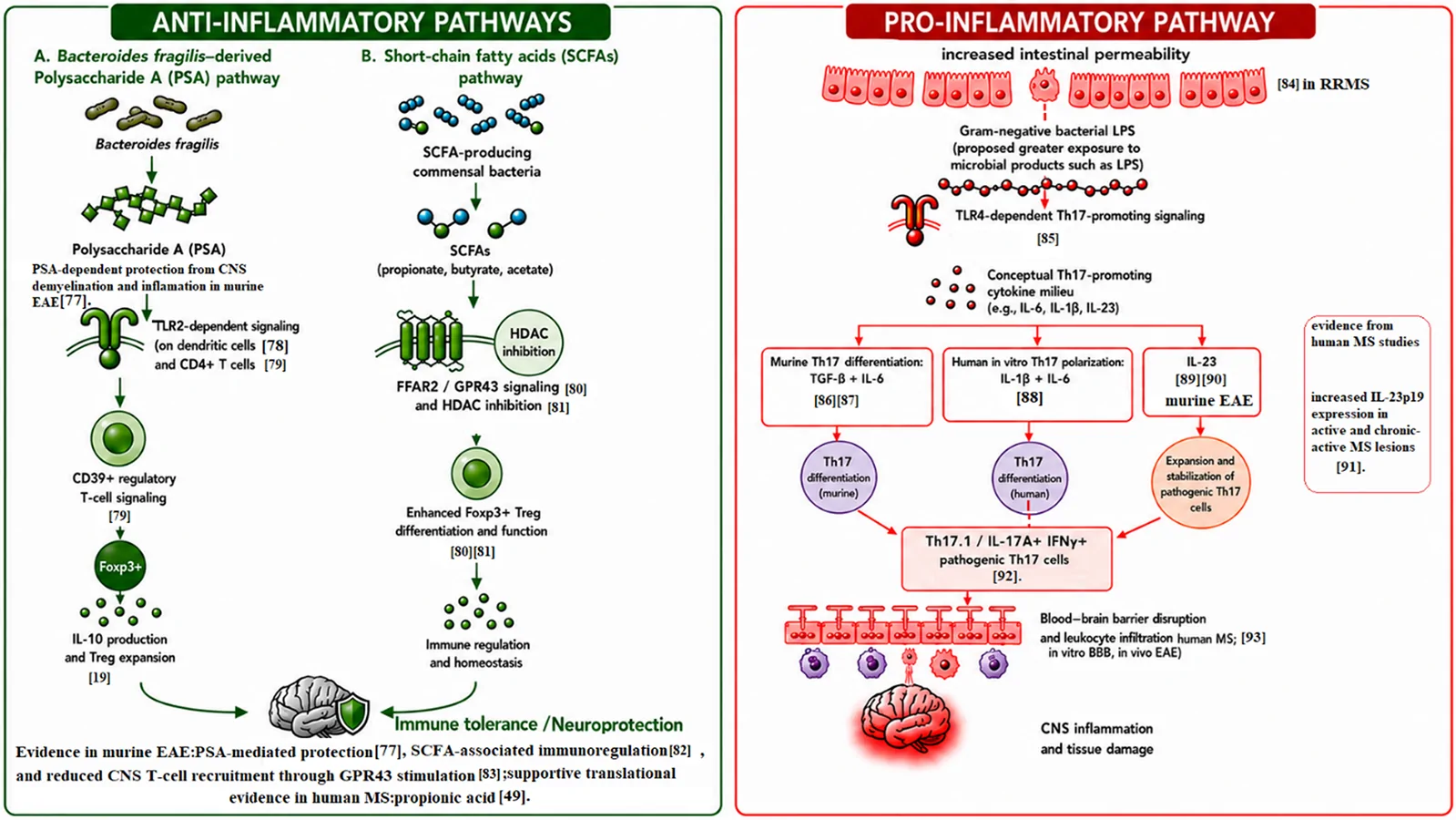
Microbiota-modulated immune pathways linking gut dysbiosis to CNS inflammation in multiple sclerosis and related autoimmune diseases. (**A**) Anti-inflammatory pathways. Two convergent commensal-derived signals promote immune tolerance. In the *Bacteroides fragilis*–polysaccharide A (PSA) pathway[23], PSA engages Toll-like receptor 2 (TLR2) on dendritic cells [24] and CD4 + T cells [25], inducing CD39 + regulatory T cells that produce interleukin-10 (IL-10) and expand Treg populations. In the short-chain fatty acid (SCFA) pathway, microbially derived SCFAs (propionate, butyrate, acetate) signal through free fatty acid receptor 2/G-protein-coupled receptor 43 (FFAR2/GPR43) [26]and inhibit histone deacetylases (HDAC) [27], enhancing FOXP3 + Treg differentiation and function. Both arms converge on immune tolerance and neuroprotection: evidence includes PSA-mediated protection in murine experimental autoimmune encephalomyelitis (EAE), SCFA-associated immunoregulation [28] and reduced CNS T-cell recruitment via GPR43 stimulation [29], with propionic acid supplementation offering supportive translational evidence in human MS. (**B**) Pro-inflammatory pathway. Dysbiosis and increased intestinal permeability, reported in relapsing-remitting MS (RRMS) [30], are proposed to increase epithelial exposure to gram-negative bacterial lipopolysaccharide (LPS). LPS activates TLR4-dependent signaling and a Th17-promoting cytokine milieu (IL-6, IL-1β, IL-23) [31]. Murine Th17 differentiation is driven by TGF-β [32]plus IL-6 [33]; human in vitro Th17 polarization is driven by IL-1β plus IL-6 [34]; IL-23 [35] expands and stabilizes pathogenic Th17 cells (shown in murine EAE) [36]. These converge on Th17.1/IL-17 A + IFN-γ + pathogenic Th17 cells, associated with BBB disruption and leukocyte infiltration [37], leading to CNS inflammation and tissue damage. Inset: human MS evidence, including increased IL-23p19 expression [38] in active and chronic-active MS lesions [39]

Dysbiosis results in a significant reduction of SCFAs—specifically butyrate, propionate, and acetate—which are essential microbial metabolites that promote Treg stability via FOXP3 upregulation, reinforce epithelial and BBB integrity by enhancing tight junction protein expression, and suppress dendritic cell driven pro-inflammatory signaling [10]. These immunoregulatory pathways mediated by microbial metabolites and bacterial molecules are illustrated in Fig. 1.

SCFAs normally maintain epithelial and endothelial resilience by regulating tight junction proteins, such as claudins and occludins, through GPR41/43 signaling. Their depletion, combined with increased levels of LPS, increases intestinal permeability (“leaky gut”) and compromises BBB integrity [40]. This structural failure allows activated Th17 lymphocytes and myeloid cells to infiltrate the CNS, where they amplify microglial activation and oxidative stress [41].

## Immunological skewing and the role of molecular mimicry

The metabolic and structural consequences of dysbiosis translate into a pro-inflammatory systemic environment. MS-associated microbial shifts promote Th1 and Th17 polarization, characterized by elevated levels of interferon-γ (IFN-γ) and interleukin-17 (IL-17), while simultaneously diminishing the secretion of anti-inflammatory cytokines like interleukin-10 (IL-10) and transforming growth factor-β (TGF-β). Accumulating evidence indicates that these immune alterations are closely linked to the metabolic consequences of dysbiosis, particularly the depletion of microbiota-derived SCFAs, which normally support Treg stability and immunoregulatory signaling [42].

Beyond metabolic signaling, certain microbes may drive CNS autoimmunity through molecular mimicry. In this model, microbial peptides with sequence or structural similarity to myelin antigens are processed and presented by antigen-presenting cells, activating cross-reactive T cells that recognize both microbial and self-epitopes. Such cross-reactivity may lower activation thresholds for autoreactive T cells and facilitate epitope spreading, sustaining inflammation even after the initial trigger is removed [43]. Candidate targets include major myelin-associated proteins such as myelin basic protein (MBP), myelin oligodendrocyte glycoprotein (MOG), and proteolipid protein (PLP), which are well-established autoantigens in MS and experimental autoimmune encephalomyelitis (EAE). Consistent with this framework, *Akkermansia* and *Acinetobacter* species isolated from MS patients have been shown to exacerbate Th17-skewed responses and disease severity in EAE models; while these findings do not independently prove molecular mimicry, they support a microbiota-driven route to autoantigen-specific immune activation when considered alongside evidence of antigenic homology and immune cross-reactivity [44]. Future studies combining peptide homology mapping with T-cell receptor (TCR) profiling and functional cross-reactivity assays are needed to establish direct mimicry links in human MS cohorts.

We emphasize that the molecular-mimicry evidence summarized above is derived entirely from EAE mouse models and in vitro cross-reactivity assays; direct demonstration of microbiota-driven molecular mimicry using patient-derived T-cell receptors and myelin-peptide homology mapping in human MS cohorts is still lacking. This mechanism should therefore be regarded as biologically plausible rather than clinically established.

**Fig. 2:**
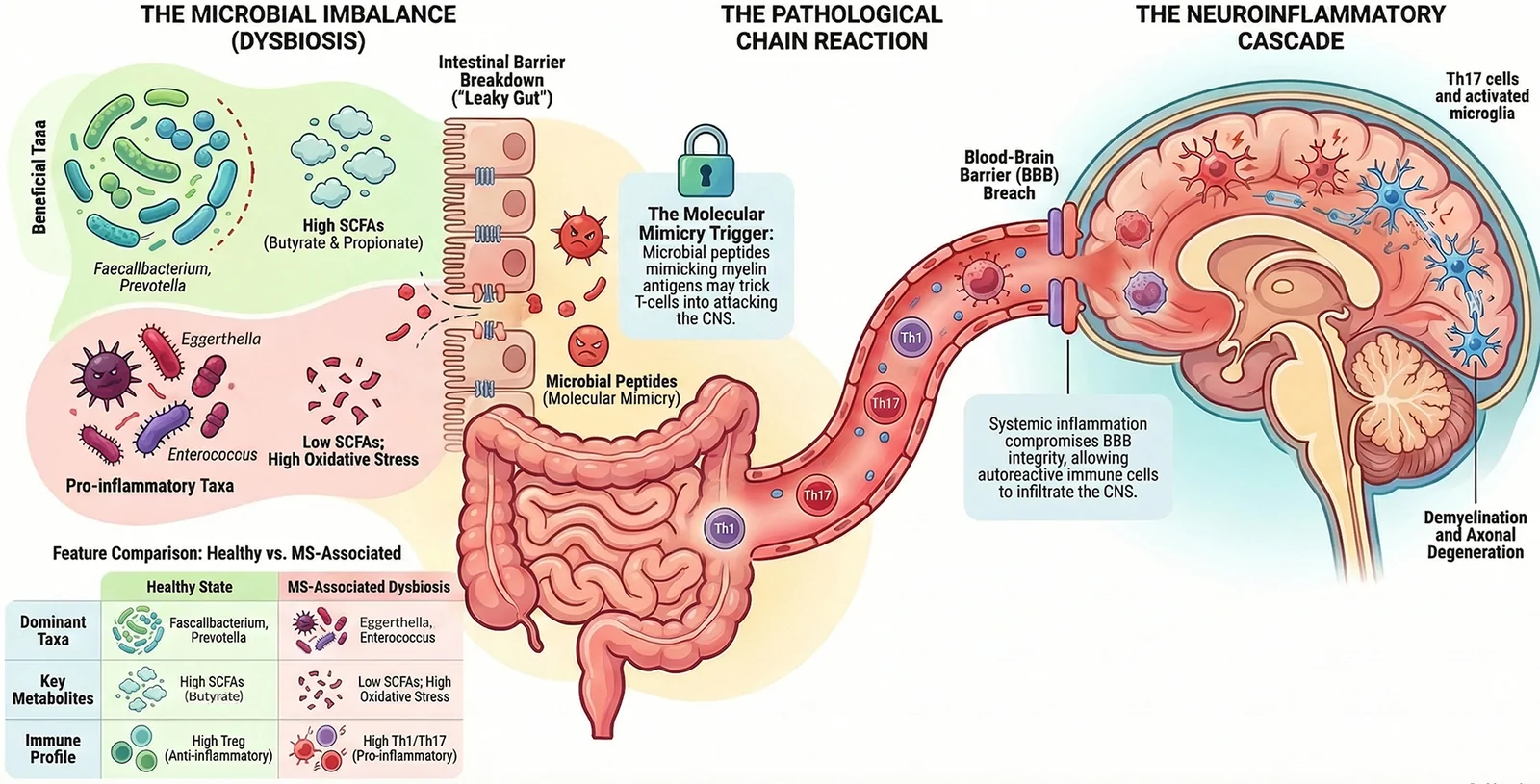
Gut–brain–immune axis in multiple sclerosis: from dysbiosis into neuroinflammation. Dysbiosis in Multiple Sclerosis is characterized by enrichment of pro-inflammatory taxa (e.g., *Enterococcus*,* and Eggerthella*) and a concurrent reduction in SCFAs due to the loss or dysfunction of SCFA-producing commensals such as Faecalibacterium. Microbiota-derived metabolites normally support regulatory immune pathways by conditioning dendritic cells and promoting Treg maintenance. In contrast, reduced SCFA availability is associated with impaired intestinal and BBB integrity, increased permeability, diminished Treg differentiation, and skewing toward pro-inflammatory Th1/Th17 responses. This immune imbalance contributes to BBB dysfunction and promotes central nervous system neuroinflammation, highlighting the interconnected gut–brain–immune axis underlying MS pathology

**Fig. 3:**
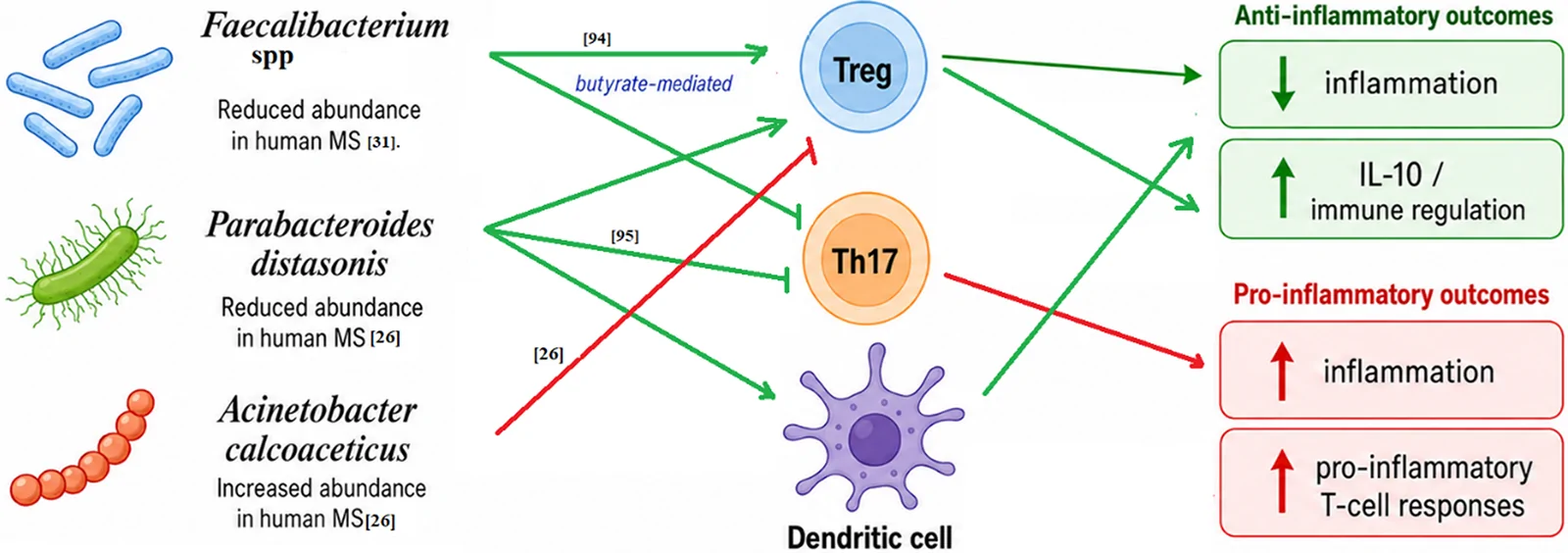
Microbiota-associated Immune Pathways Relevant to Multiple Sclerosis. *Faecalibacterium* spp. and *Parabacteroides distasonis* are reduced, whereas *Acinetobacter calcoaceticus* is increased in MS. *Faecalibacterium*-derived butyrate [46] and *P. distasonis* promote Treg-associated anti-inflammatory responses and suppress Th17-associated inflammation [47]; *P. distasonis* also modulates dendritic-cell pathways, while *A. calcoaceticus* inhibits Treg responses and promotes pro-inflammatory T-cell activity. Green arrows indicate anti-inflammatory effects, red arrows indicate pro-inflammatory effects, and blunt-ended lines indicate inhibition

Collectively, these findings support a multifaceted model in which gut dysbiosis contributes to MS pathogenesis through metabolic, immunological, and structural mechanisms. Altered microbial composition reduces SCFA production, disrupts mucosal and endothelial barriers, skews T cell responses, and promotes molecular mimicry [11]. Rather than acting in isolation, these pathways reinforce one another in a self-sustaining feedback loop, wherein immune activation further perturbs microbial composition and metabolic output, perpetuating barrier dysfunction and CNS inflammation. These interconnected pathways are illustrated in Fig. 2, which summarizes the cascade of events leading from dysbiosis to immune dysregulation and CNS inflammation in MS. Importantly, the impact of these mechanisms differs by disease stage. In RRMS, transient dysbiosis may amplify immune activation and acute barrier disruption, contributing to relapses. In contrast, progressive forms (SPMS and PPMS) exhibit sustained dysbiosis, chronic microglial activation, and cumulative neurodegeneration. These stage-specific differences highlight the need to consider both temporal and regional influences when studying the microbiota MS link [45]. Figure 3 illustrates the immunological consequences of key microbial taxa in the Egyptian cohort and their role in modulating Treg/Th17 balance and CNS inflammation.

## Taxa-level changes (global & regional comparison)

**Fig. 4:**
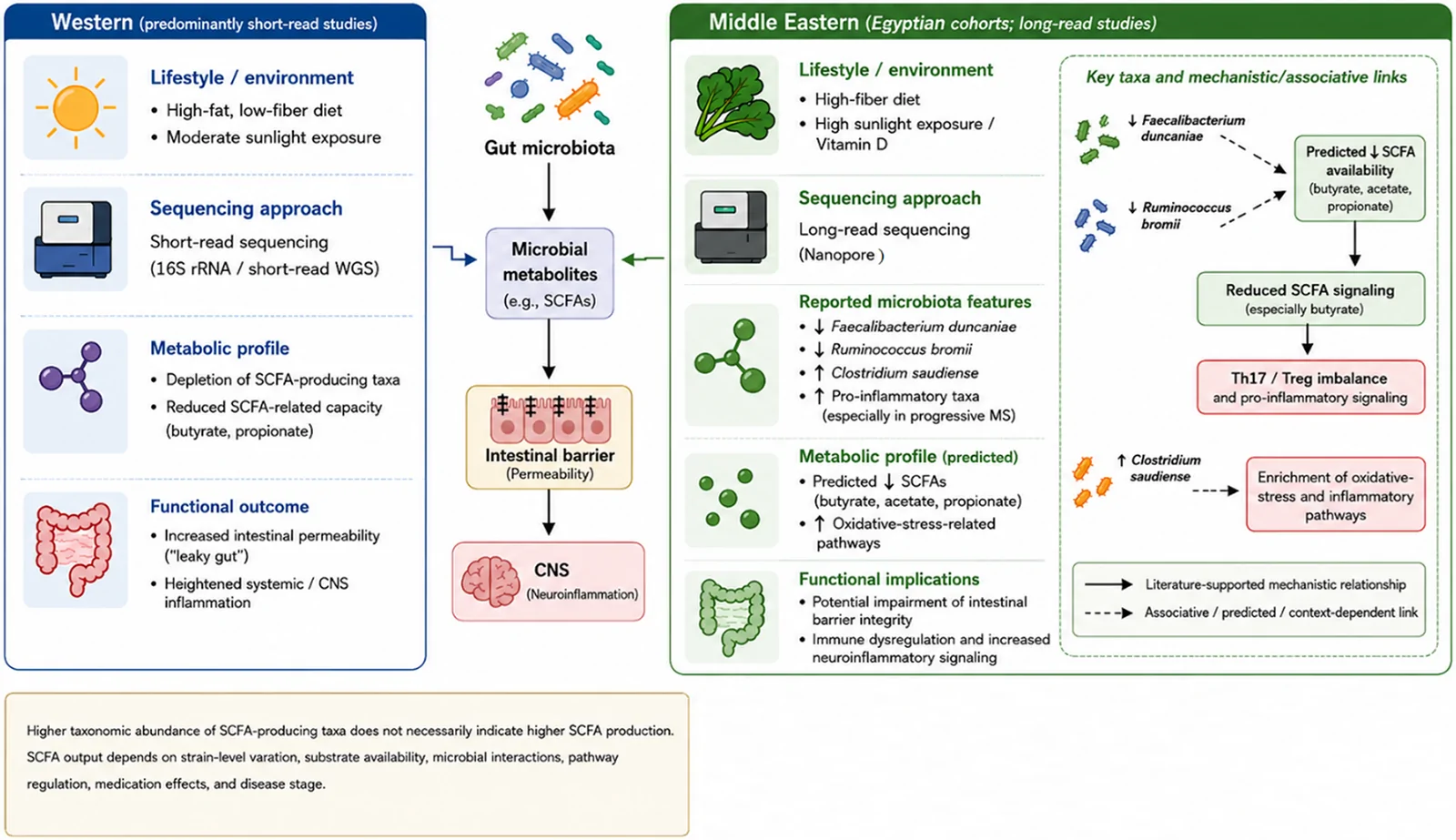
Comparative summary of gut microbiota composition and functional metabolites in MS across Western and Middle Eastern (Egyptian) cohorts. Western studies, predominantly based on short-read 16 S rRNA or short-read metagenomic sequencing, commonly report depletion of selected SCFA-producing taxa, reduced SCFA-related metabolic potential, increased intestinal permeability, and enhanced inflammatory signaling. In the Egyptian cohort using Oxford Nanopore full-length 16 S rRNA sequencing, *Faecalibacterium duncaniae* and *Ruminococcus bromii* were depleted, whereas *Clostridium saudiense* and other pro-inflammatory taxa were enriched, particularly in progressive MS. Functional prediction suggested potentially reduced SCFA-related capacity and enrichment of oxidative-stress- and immune-dysregulation-related pathways; these functional alterations were computationally inferred rather than directly measured. Such changes may contribute to impaired intestinal barrier integrity and neuroinflammatory signaling. Dietary patterns and sunlight/vitamin D exposure are presented as regional contextual factors and were not uniformly measured across all cohorts. Taxonomic abundance does not necessarily reflect metabolite production, which may also depend on strain-level variation, substrate availability, microbial interactions, pathway activity, medication exposure, and disease stage. Solid arrows indicate literature-supported mechanistic relationships, whereas dashed arrows indicate cohort associations or predicted/context-dependent links

Regional factors, including diet, genetics, and lifestyle, further shape the gut microbiota and its interactions with the CNS. Egyptian cohorts, for example, combine high fiber diets with greater sunlight exposure but still demonstrate SCFA depletion and enrichment of oxidative stress pathways not observed in Western populations. Such findings underscore the value of region-specific studies for uncovering localized disease drivers and identifying culturally tailored therapeutic strategies Fig. 4.

MS-associated gut microbiota alterations show a broadly shared dysbiosis pattern across cohorts, although the specific taxa differ by region [48, 49]. Western studies commonly report depletion of SCFA-producing taxa, particularly *Faecalibacterium* and *Prevotella*, together with altered *Bacteroides: Prevotella* ratios and shifts in Clostridial metabolism [50]. In Egypt, long-read Oxford Nanopore sequencing revealed enrichment of pro-inflammatory taxa, including *Enterococcus faecium* and *Clostridium saudiense*, with depletion of *Faecalibacterium duncaniae* and *Ruminococcus bromii*; functional predictions further indicated oxidative stress– and neurotransmitter-related signatures, along with reduced butyrate-associated metabolism [9]. A complementary qPCR-based study in Egyptian RRMS cohort reported increased *Actinobacteria*, *Desulfovibrio*, and lactic acid bacteria, alongside reduced Clostridium cluster IV [51]. In that cohort, the most internally consistent clinical association was for *Bifidobacterium*, which was higher in patients with lower disability (EDSS ≤ 3.5 vs. > 3.5, *P* = 0.02) and inversely correlated with EDSS score (*P* = 0.04; Fig. 3 of ref [45]), suggesting a possible protective role. By contrast, *Akkermansia* showed no case–control difference and only a marginal inverse correlation with disease duration (*r* = − 0.33, *P* = 0.04) and should therefore be interpreted with caution.

In Iran, a primary case–control study using targeted qPCR reported reduced *Bacteroides fragilis* and *Bifidobacterium* spp. (with lower, non-significant *Faecalibacterium prausnitzii*) in treatment-naïve MS patients [52], consistent with loss of PSA- and butyrate-associated immunoregulatory commensals; whole-community sequencing and metabolomic data from Iranian cohorts remain limited. In India, a North Indian case–control study using 16 S rRNA (Illumina MiSeq) sequencing found reduced alpha diversity with depletion of *Faecalibacterium prausnitzii*, *Monoglobus pectinilyticus*, and *Bacillus* species, together with elevated serum zonulin indicating increased intestinal permeability and a predicted shift from short-chain toward long-chain fatty-acid metabolism [53]; species-level data from the region nonetheless remain limited. Because the Egyptian, Iranian, and Indian datasets differ substantially in cohort size (as few as 33–84 MS patients per study), sequencing platform (long-read Nanopore, targeted qPCR, and short-read 16 S, respectively), and depth of taxonomic resolution, the cross-regional differences summarized above should not be interpreted as evidence of true biological divergence between populations. Harmonized, adequately powered, multi-platform studies are needed before region-specific dysbiosis signatures can be treated as biologically distinct entities rather than artifacts of methodology and sample size. Overall, current evidence supports region-specific microbial and functional signatures within a broadly shared dysbiosis framework in MS. Key taxa-level differences across regions are summarized in Table 1.

**Table 1:** Cross regional comparison of microbial alterations in MS, including cohort size, detection methodology, and level of evidence

Region / cohort	Study (ref.)	Sample size (MS / controls)	Sequencing / detection technology	Key altered taxa	Functional / unique patterns	Evidence status	Evidence gaps
**Global (meta-analysis)**	Lin et al. 2024 [50]; Zhang et al. 2024 [54]	Lin: 524 (257 MS / 267 HC), 7 cohorts. Zhang: 2,885 participants, 26 studies	Meta-analyses of 16 S rRNA datasets	↓ Faecalibacterium prausnitzii; ↓ Prevotella copri	Altered Bacteroides: Prevotella ratio; consistent SCFA depletion	Replicated — pooled across multiple independent cohorts	Non-Western cohorts underrepresented; genus/16S-level resolution
**Western (USA / Europe / Japan)**	Miyake 2015 [55]; Jangi 2016[7]; Ventura 2019 [49]; Cox 2021 [8]	Miyake 20/40; Jangi 60/43; Ventura 45/44 (16 S) + 24/24 (shotgun); Cox progressive-MS cohort	16 S rRNA (pyrosequencing / Illumina); shotgun metagenomics in subset	↑ Actinomyces; ↑ Akkermansia muciniphila; ↑ Blautia; variable Clostridium	Functional shifts in Clostridial metabolism; ethnicity-independent Clostridium enrichment	Replicated — concordant across several independent cohorts	Cohort heterogeneity; functional validation needed
**Egypt (targeted molecular)**	Elgendy et al. 2021[51]; Mekky et al. 2022 [56]	Elgendy: 40 RRMS / 22 HC. Mekky: 30 RRMS / 20 HC	Targeted real-time qPCR (16 S rRNA group-specific primers) — not sequencing-based	↑ Actinobacteria; ↑ Desulfovibrio; ↑ lactic-acid bacteria; ↓ Clostridium cluster IV	Inverse correlation between Bifidobacterium and EDSS score	Preliminary — small single-centre cohorts; genus-level qPCR	Small cohort; genus-level; no sequencing / functional profiling
**Egypt (long-read sequencing)**	Alshinnawy et al. 2025 [9]	33 MS (20 RRMS, 13 PMS) / 10 HC	Full-length 16 S rRNA, Oxford Nanopore long-read sequencing	↑ Enterococcus faecium; ↑ Romboutsia timonensis; ↑ Clostridium saudiense; ↓ Faecalibacterium duncaniae; ↓ Ruminococcus bromii	↑ oxidative-stress and neurotransmitter pathways; ↓ butyrate metabolism (predicted)	Preliminary — single-centre pilot; species-level resolution; functional inference only	Pilot cohort; independent replication pending
**Iran**	[52]	36 / 36 (age–sex-matched; design-powered at 36 per group)	Targeted qPCR (relative/CFU-based abundance of selected taxa)	↓ Bacteroides fragilis; ↓ Bifidobacterium spp. (both significant); ↓ Faecalibacterium prausnitzii (non-significant)	Loss of PSA-producing *B. fragilis* and butyrogenic *Bifidobacterium* → reduced immunoregulatory/SCFA-associated capacity; captures signal before DMT confounding	Primary case–control; preliminary (single-centre, targeted qPCR)	Only three candidate taxa assayed; no whole-community sequencing or metabolomics; correlational (no longitudinal/causal data)
**India** (North India; RRMS, EDSS ≤ 5.5)	[53]	84 RRMS / 106 HC	16 S rRNA (Illumina MiSeq)	↓ *Faecalibacterium prausnitzii*, *Monoglobus pectinilyticus*, *Bacillus* spp. (with reduced Lachnospiraceae/Oscillospiraceae/*Parasutterella*; ↑ *Lachnospiraceae* UCG-008; Firmicutes enriched at phylum level)	↓ alpha diversity; elevated serum zonulin (↑ intestinal permeability) **without** raised calprotectin; predicted shift toward long-chain-fatty-acid biosynthesis over SCFA generation → pro-inflammatory/leaky-gut phenotype	Primary case–control	Metabolic pathways inferred (predicted, not measured metabolomics); cross-sectional; single-region (North India)

Abbreviations: MS, multiple sclerosis; RRMS, relapsing–remitting MS; PMS, progressive MS; HC, healthy controls; SCFA, short-chain fatty acid; qPCR, quantitative polymerase chain reaction; 16 S rRNA, 16 S ribosomal RNA gene

Evidence status: “Replicated” denotes findings concordant across two or more independent cohorts or confirmed by pooled meta-analysis; “Preliminary” denotes single-centre, pilot-scale, or genus-level findings not yet independently reproduced; “indirect” denotes patterns inferred from regional or secondary sources rather than a dedicated primary cohort

Note: Where a primary cohort with a reported sample size was not available (Iran, India), this is stated explicitly rather than estimated. Arrows indicate reported enrichment (↑) or depletion (↓) relative to controls. Cross-row comparison should account for differences in sequencing technology and taxonomic resolution

## Sequencing technologies in MS microbiome research: challenges and considerations

Accurate interpretation of gut microbiome alterations in multiple sclerosis (MS) critically depends on sequencing methodology, as taxonomic resolution and functional inference vary substantially across platforms. Earlier MS microbiome studies relied predominantly on 16 S rRNA gene sequencing, which enables genus-level profiling but limits species-level resolution and direct functional interpretation of immune-relevant pathways [57]. The evolution of these platforms has markedly improved species-level classification and pathway prediction, ultimately refining biomarker discovery and therapeutic potential.

Consequently, reported differences across studies and particularly across geographic regions may reflect methodological heterogeneity rather than true biological divergence. Recent adoption of shotgun metagenomics and long-read sequencing technologies has substantially improved species- and strain-level resolution and enabled more reliable characterization of microbial functions linked to immune regulation, including SCFA biosynthesis, oxidative stress pathways, and neurotransmitter and tryptophan metabolism [7, 55]. These advances are especially relevant for cross-regional analyses, as distinct dietary, environmental, and genetic contexts in underrepresented populations may shape microbial function independently of taxonomic composition. Integrating high-resolution sequencing with functional annotation and immunophenotyping is therefore essential to harmonize global MS microbiome studies and to translate region-specific microbial signatures into mechanistically informed biomarkers and therapeutic strategies [58, 59].

Recent comparative analyses across global cohorts underscore the profound impact of sequencing methodology on taxonomic resolution and the subsequent biological interpretation of MS-associated dysbiosis. High-resolution profiling reveals that contradictions in the literature are often artifacts of genus-level 16 S sequencing, which masks critical species-specific signatures. As detailed in Table 2, studies employing whole-genome shotgun metagenomics consistently demonstrate superior discriminatory power, enabling strain- and gene-level characterization of microbial communities and the identification of functional pathways directly linked to immune modulation, short-chain fatty acid metabolism, and pro-inflammatory signaling. In contrast, amplicon-based approaches frequently collapse heterogeneous taxa into single genera, obscuring biologically meaningful divergence among closely related species with opposing immunological roles. These methodological disparities not only complicate cross-study comparisons but also contribute to inconsistent reports of enrichment or depletion for key taxa in MS cohorts. Collectively, the evidence suggests that resolving MS-associated microbial signatures—and translating them into mechanistic insight—requires integrative, high-resolution multi-omics frameworks that couple deep metagenomic sequencing with host immune, metabolic, and clinical metadata.

**Table 2:** Functional and taxonomic comparison of MS-associated gut microbiota across global studies using different sequencing technologies

Study	Region / cohort	Technology	Study type	Main MS-associated findings	Functional / translational note
Miyake et al. (2015) [55]	Japan	16 S rRNA pyrosequencing	Human case-control	Depletion of bacteria belonging to Clostridia XIVa and IV clusters in MS.	Supports loss of taxa linked to immunoregulatory and butyrate-associated functions.
Jangi et al. (2016) [7]	USA	16 S rRNA	Human case-control	Increased Methanobrevibacter and Akkermansia, with decreased Butyricimonas in MS.	Linked microbial shifts to immune-related pathways, including dendritic-cell maturation, IFN signaling, and NF-kB signaling.
Cekanaviciute et al. (2018) [13]	USA	16 S rRNA (spore-forming fraction) + immune functional assays	Human case-control / mechanistic	MS-associated alterations in the spore-forming bacterial fraction.	Connected taxonomic changes to altered T-cell responses and immune function.
Ventura et al. (2019) [49]	USA, treatment-naive multi-ethnic cohort	16 S rRNA	Human case-control	Broad microbiome differences early in disease; reported increases in taxa such as Akkermansia and Blautia, with shifts in Clostridium across ethnic groups.	Important because findings were assessed across different ethnic backgrounds.
Cox et al. (2021) [8]	USA, progressive MS	16 S-based microbiome profiling	Human case-control	Progressive MS showed a distinct gut microbiome profile relative to controls.	Supports disease-course-specific dysbiosis in progressive MS.
Cantoni et al. (2022) [60]	Multi-omic MS cohort	Shotgun metagenomics + immune/metabolome integration	Human case-control	Multi-system alterations in gut microbiota, immune features, and blood metabolome in MS.	Illustrates the functional depth added by shotgun metagenomics.
Zhou et al. (2023) [61]	MS patients and paired controls	Shotgun metagenomics	Human case-control	Distinct microbial networks associated with untreated MS and disease course.	Useful high-resolution metagenomic study with host-environment control design.
Thirion et al. (2023) [62]	Prospectively collected MS cohort	Shotgun-sequencing-based gut metagenome analysis	Human cohort study	Gut microbiota varied with disease course and inflammatory context.	Good example of sequencing depth for disease-activity associations.
Zhang et al. (2024) [54]	Global 16 S literature	Meta-analysis of 16 S rRNA studies	Review / meta-analysis	Included 26 studies and 2,885 participants; no overall significant alpha-diversity difference, but lower Shannon diversity in active MS; compositional shifts may be more informative than diversity alone.	Most comprehensive meta-analytic synthesis of the 16 S literature base to date; not a primary cohort study.
Alshinnawy et al. (2025) [9]	Egypt	Long-read Oxford Nanopore sequencing	Human case-control / subtype-focused	Enrichment of Enterococcus faecium, Clostridium saudiense, and Romboutsia timonensis with depletion of Faecalibacterium duncaniae and Ruminococcus bromii.	Long-read sequencing improved species-level resolution and linked taxa to oxidative stress and neurotransmitter-related signatures.

To overcome the limitations of individual methods, hybrid sequencing strategies are increasingly recommended. Long-read 16 S sequencing provides robust species-level resolution, while shotgun metagenomics delivers functional insights. These methods can be further validated using targeted approaches such as quantitative PCR, metabolomics (including SCFA profiling), and cytokine assays. Future directions include high-fidelity (HiFi) PacBio sequencing, metatranscriptomics, and multi-omics frameworks that integrate microbiome data with host gene expression, proteomics, and metabolomics. The comparative strengths and limitations of long-read and short-read metagenomic platforms for microbial community profiling have been reviewed in detail [63]. These combined strategies will provide a more comprehensive understanding of the gut–brain axis and its role in MS pathogenesis.

## Microbiota-targeted therapeutic strategies in multiple sclerosis

Growing evidence suggests that modulation of the gut microbiota may influence immune dysregulation, intestinal barrier integrity, microbial metabolite production, and disease-related inflammatory activity in MS. The gut microbiota can affect systemic- and CNS-directed immunity through interactions with T cells, B cells, antigen-presenting cells, intestinal barrier pathways, microbial metabolites, and neuroimmune signaling [64]. However, microbiota-targeted interventions in MS remain largely investigational and should currently be considered adjunctive or experimental rather than established disease-modifying therapies. Table 3 summarizes the strategies for Microbiota-targeted therapies in MS including dietary modulation, short-chain fatty acid supplementation, probiotics, prebiotics, synbiotics, fecal microbiota transplantation, and other ecosystem-level approaches [65]. Large, controlled human studies are still needed before these interventions can be recommended as routine MS therapy [66]. To state this explicitly: probiotics, prebiotics, short-chain fatty acid supplementation, and fecal microbiota transplantation have not been shown in adequately powered, randomized controlled trials to alter relapse rate, disability progression, or MRI outcomes in MS. None of these approaches is currently approved or recommended as a disease-modifying therapy, and they should not be substituted for established MS treatments.

### Metabolite- and diet-based interventions

Dietary modulation and supplementation with microbiota-derived metabolites aim to restore immunoregulatory signaling that may be disrupted in MS. SCFAs, especially acetate, propionate, and butyrate, are produced by bacterial fermentation of dietary fiber and can influence immune tolerance, intestinal barrier function, and inflammatory T-cell differentiation [67]. Butyrate-producing bacteria and related metabolites have been reported to be reduced in MS cohorts, supporting the hypothesis that altered microbial metabolism may contribute to immune dysregulation.

Among SCFA-based interventions, propionic acid has the strongest early human evidence. Duscha et al. reported that propionic acid supplementation in MS was associated with increased functional regulatory T cells, reduced Th1/Th17 activity, and post-hoc associations with reduced annual relapse rate, disability stabilization, and reduced brain atrophy after long-term intake. However, these findings should be interpreted cautiously because post-hoc clinical outcomes require confirmation in larger randomized controlled trials [68].

Dietary strategies are broader than SCFA supplementation. High-fiber and Mediterranean-style dietary patterns may support microbial diversity and SCFA production. A 2024 pediatric-onset MS study reported that higher Mediterranean diet score and higher fiber intake were associated with lower odds of MS, with gut microbiota features potentially mediating part of this association [69]. However, this evidence is observational and does not prove therapeutic efficacy in established MS.

Other dietary approaches, such as ketogenic diets and intermittent fasting, have also been explored [70]. Ketogenic diets appear feasible and tolerable in MS and may improve patient-reported outcomes in some studies, while intermittent fasting has been suggested to affect immune and microbiome-related pathways. Nevertheless, clinical evidence remains limited, heterogeneous, and not yet sufficient to define these diets as microbiota-directed MS treatments.

### Probiotics and live biotherapeutic products

Probiotics, prebiotics, and synbiotics have been explored as adjunctive strategies to modulate dysbiotic microbial communities, microbial metabolite production, intestinal barrier integrity, and systemic immune responses in MS [71]. Small human trials suggest that selected strain-defined formulations may influence inflammatory cytokines, Treg-associated markers, oxidative stress, and patient-reported symptoms [72]; However, current evidence is still preliminary and does not support probiotics as disease-modifying therapy.

The most relevant human probiotic studies in MS have used multi-strain formulations containing combinations of *Lactobacillus*,* Bifidobacterium*,* Streptococcus thermophilus*,* Lactococcus lactis*,* Bacillus subtilis*, and, in one trial, the yeast probiotic *Saccharomyces boulardii*.

Tankou et al. investigated VSL#3 (high-potency multi-strain probiotic formulation) in healthy controls and MS patients. The study used a high-dose probiotic formulation enriched in *Lactobacillus*,* Streptococcus*, and *Bifidobacterium* species. In MS participants, VSL#3 administration was associated with microbiome shifts and anti-inflammatory innate immune changes, including reduced intermediate monocytes and reduced HLA-DR expression on myeloid dendritic cells. Importantly, the microbiome changes returned toward baseline after probiotic discontinuation, suggesting that effects may not persist without continued exposure [73].

Rahimlou et al. conducted a 12-week randomized double-blind trial using a 14-strain probiotic formulation. The strains included *Bacillus subtilis PXN21*,* Bifidobacterium bifidum PXN23*,* B. breve PXN25*,* B. infantis PXN27*,* B. longum PXN30*,* Lactobacillus acidophilus PXN35*,* L. delbrueckii ssp. bulgaricus PXN39*,* L. casei PXN37*,* L. plantarum PXN47*,* L. rhamnosus PXN54*,* L. helveticus PXN45*,* L. salivarius PXN57*,* Lactococcus lactis ssp. lactis PXN63*, and *Streptococcus thermophilus PXN66*. Compared with placebo, probiotic supplementation reduced CRP, TNF-α, and IFN-γ and increased FOXP3 and TGF-β, suggesting a shift toward anti-inflammatory and regulatory immune activity [74].

In a separate long-term (six-month) trial using a comparable multi-strain formulation, the same group further reported that probiotic supplementation was associated with increased BDNF, reduced IL-6, and improvements in general health, depression, fatigue, and pain scores. However, these findings mainly support symptom and biomarker improvement, rather than definitive evidence for relapse prevention or long-term disability modification [75].

Asghari et al. evaluated Saccharomyces boulardii in a randomized clinical trial of 40 MS patients over four months. The intervention significantly reduced hs-CRP increased total antioxidant capacity, and improved pain, fatigue, and some quality-of-life measures. No serious adverse effects or relapses were reported during the trial, but the study was small and did not include detailed stool microbiome confirmation [76].

Overall, probiotic interventions show immunomodulatory potential in MS, but reported effects remain inconsistent because studies differ in strain composition, dose, duration, participant characteristics, background disease-modifying therapies, and outcome measures. Therefore, the field should move from empirical multi-strain supplementation toward strain-defined, functionally validated live biotherapeutic products with reproducible immunological mechanisms [77].

### Prebiotics and synbiotics

Prebiotics are fermentable substrates that selectively support beneficial microbial growth and SCFA production, while synbiotics combine probiotics with prebiotics. In MS, these approaches are attractive because they may support endogenous anti-inflammatory microbial metabolism rather than only adding exogenous organisms [78].

Farber et al. conducted a randomized cross-over trial comparing Visbiome probiotic supplementation with Prebiotin, an oligofructose-enriched inulin prebiotic, in people with relapsing-remitting MS receiving B-cell depletion therapy. Both interventions were reasonably well tolerated, with mostly mild adverse events. Probiotics improved bowel control over six weeks, but broader patient-reported outcomes did not clearly improve in that short time frame [79] .

Synbiotic approaches have also been tested in progressive MS. Moravejolahkami et al. studied an anti-inflammatory/antioxidant-rich diet combined with synbiotic supplementation in progressive MS. The intervention significantly reduced fecal calprotectin, a marker of intestinal inflammation, and improved some clinical manifestations. However, because the intervention combined diet and synbiotics, the independent effect of the synbiotic product cannot be isolated [80].

### Fecal microbiota transplantation and ecosystem-level replacement

FMT is a more comprehensive ecosystem-level strategy designed to restructure the gut microbial community. Unlike probiotics, which introduce selected strains, FMT transfers a complex donor microbial ecosystem. This may theoretically correct broader dysbiosis, but it also introduces greater safety, donor-screening, reproducibility, and regulatory challenges [81].

Al et al. conducted a pilot randomized controlled trial of FMT in relapsing-remitting MS. Monthly FMT was administered for up to six months. The study reported that FMT was safe and tolerable in this small cohort, was associated with donor-specific microbiota shifts, and may improve elevated small intestinal permeability. However, the sample size was very small, and the study was not powered to establish clinical efficacy [82].

Recent case-report evidence has also suggested that capsule-based FMT may be feasible and well tolerated in MS, with reported gastrointestinal and neurological improvement in an individual patient [83]. However, case reports cannot establish efficacy, Therefore, FMT is still experimental in MS and requires larger controlled trials to confirm safety and efficacy.

### Evidence gaps, translational challenges, and future directions

Despite strong mechanistic rationale, the main limitation across microbiota-targeted therapies in MS is the lack of robust clinical validation. Most available studies are small, short-term, and heterogeneous. Many rely on patient-reported symptoms, inflammatory biomarkers, or microbiome composition changes rather than strong clinical endpoints such as annualized relapse rate, confirmed disability progression, MRI lesion activity, brain atrophy, or neurodegeneration markers [84].

Interpretation is further complicated by major confounding factors, including baseline microbiome variability, diet, geography, ethnicity, antibiotic exposure, BMI, disease phenotype, disease stage, and background disease-modifying therapies. DMTs and diet can themselves alter gut microbial composition and immune responses, making it difficult to separate whether microbiome changes are caused by the intervention, the disease, or the treatment background [85].

Another major challenge is reproducibility. Microbiome studies differ in stool collection methods, sequencing platforms, bioinformatic pipelines, taxonomic classification, metabolomic analysis, and statistical correction methods. In addition, many probiotic studies use different strains, doses, CFU counts, treatment durations, and outcome measures, which prevents direct comparison across trials [86].

Future studies should prioritize randomized placebo-controlled designs, strain-defined interventions, standardized CFU dosing, controlled dietary assessment, longitudinal stool metagenomics, metabolomics, immune-cell phenotyping, intestinal permeability markers, MRI outcomes, relapse outcomes, disability progression, and long-term safety monitoring. To enable comparison across trials, we recommend that microbiota-targeted MS studies adopt a harmonized core outcome set anchored on the annualized relapse rate and confirmed disability progression (sustained EDSS change) as primary clinical endpoints, with MRI activity (new/enlarging T2 and gadolinium-enhancing lesions and brain-atrophy measures) as key imaging endpoints, and treat cytokine, SCFA, and microbiome-composition changes as supportive mechanistic (not primary) outcomes. Equally important, dietary intake should be captured with validated, standardized instruments (for example, food-frequency questionnaires with fiber and Mediterranean-diet indices) and participants followed longitudinally, so that diet and time are modelled as covariates rather than left as residual confounders.

Patient stratification will also be essential. Microbiota-targeted therapies may be more effective in specific subgroups, such as patients with baseline dysbiosis, low SCFA-producing bacteria, impaired gut-barrier function, high inflammatory activity, specific dietary patterns, or particular DMT exposure. This personalized approach may help identify which patients are most likely to benefit from microbiota-based interventions [87]. In summary, despite encouraging mechanistic and early clinical signals, probiotics, SCFA supplementation, prebiotics/synbiotics, and FMT remain experimental adjuncts in MS. None has yet demonstrated disease-modifying efficacy in adequately powered randomized trials, and their use outside research settings should not be regarded as a substitute for approved disease-modifying therapies.

**Table 3:** Microbiota-targeted therapeutic strategies in MS

Therapeutic strategy	Examples / intervention	Mechanism / target	Study design / population	Key findings / outcome	Evidence level	Pros	Limitations	References
Probiotics / live biotherapeutics	Multi-strain formulations including *Lactobacillus*,* Bifidobacterium*,* Streptococcus thermophilus*,* Lactococcus lactis*,* Bacillus subtilis*, and *Saccharomyces boulardii*	Modulate dysbiotic microbial communities; improve gut barrier function; influence cytokine profiles; ↑ Treg-related responses; ↓ Th1/Th17 inflammatory activity; may support SCFA-related pathways	Small randomized trials and pilot studies in MS patients, including studies from Iran and other clinical cohorts	reduced inflammatory markers such as CRP, TNF-α, IFN-γ, and IL-6; increased FOXP3/TGF-β; improved fatigue, pain, depression, oxidative stress, and quality-of-life measures	Low to moderate: mostly small RCTs and pilot studies	Low risk, scalable, strain-customizable, relatively easy to administer	Effects are strain-specific; variable engraftment; short duration; small sample sizes; limited evidence for relapse or disability improvement	[73, 75, 76]
Prebiotics / synbiotics	Prebiotin / oligofructose-enriched inulin; synbiotic supplementation combined with anti-inflammatory or antioxidant-rich diet	Promote beneficial microbial growth; increase endogenous SCFA production; support anti-inflammatory microbial metabolism; may reduce gut inflammation	Randomized cross-over trial in RRMS patients receiving B-cell depletion therapy; randomized trials in progressive MS using diet + synbiotics	probiotics improved bowel control in one cross-over study; synbiotic + diet reduced fecal calprotectin and improved some clinical manifestations	Preliminary to moderate: small clinical trials, often combined with diet	Supports endogenous microbiota; may be safer and more acceptable than FMT; can be integrated with diet	Difficult to separate effect of synbiotic from diet; adherence issues; indirect mechanism; limited MS-specific clinical endpoints	[79, 80]
Fecal microbiota transplantation (FMT)	Donor fecal microbiota transplantation; capsule-based FMT in case reports	Restores or restructures the whole gut microbial ecosystem; may improve microbial diversity and intestinal barrier integrity	EAE models; pilot randomized controlled trial and case reports in MS patients, mainly Western cohorts	Pilot data suggest FMT is safe and tolerable, may reduce intestinal permeability, and induces donor-specific microbiota shifts; symptom benefit remains anecdotal.	Low / experimental: pilot studies and case reports	Broad ecosystem-level effect; potential durable microbial restructuring	Risk of pathogen transfer; donor-screening complexity; regulatory challenges; cost; optimal route/dose unknown; clinical efficacy unproven	[82, 83]
SCFA supplementation	Propionic acid; butyrate-related approaches	Restores SCFA-related immunometabolic signaling; ↑ Treg function; ↓ Th1/Th17 inflammatory activity; supports gut barrier and anti-inflammatory immune regulation	Human interventional study in MS patients in Germany; supported by EAE animal models	Propionic acid supplementation increased functional Tregs, reduced Th1/Th17 cells, and was associated in post-hoc analyses with reduced annual relapse rate, disability stabilization, and reduced brain atrophy	Preliminary but promising: mechanistic human evidence plus animal support	Targeted mechanism; strong biological rationale; easier to standardize than whole microbiome replacement	Optimal dose, duration, and long-term safety unclear; clinical outcomes require larger RCT confirmation	[68]
Dietary interventions	High-fiber diet; Mediterranean-style diet; ketogenic diet; intermittent fasting	Supports SCFA-producing bacteria; alters endogenous microbiota and microbial metabolites; may reduce inflammatory signaling and improve metabolic status	Observational cohorts, pediatric-onset MS case-control study, small dietary trials, and EAE models	Higher Mediterranean-diet and fiber intake correlated with lower pediatric-onset MS odds; ketogenic and fasting diets may affect selected symptoms or biomarkers, but causality and disease-modifying effects are unproven	Low to moderate: observational and small interventional studies	Natural, sustainable, lifestyle-integrated, low cost, can complement standard MS therapy	Poor adherence; indirect effects; dietary confounding; difficult to standardize; not proven as standalone treatment	[69, 88–91]

## Sex-based microbiome differences in MS

Sex differences are a defining feature of multiple sclerosis (MS), with higher disease incidence in females and sex-dependent variation in immune responses and disease course. Emerging evidence suggests that sex-specific interactions between host hormones, immune signaling, and the gut microbiota may contribute to these disparities. Estrogens and androgens modulate both innate and adaptive immunity, influencing Treg function, Th17 polarization, and cytokine production, which in turn shape microbial composition and function [92, 93]. Experimental studies demonstrate that estrogen signaling can promote immunoregulatory pathways, including enhanced Treg differentiation and suppression of proinflammatory Th17 responses, effects that are partially microbiota-dependent [58]. Conversely, androgen signaling has been linked to distinct microbial profiles and reduced inflammatory susceptibility in male models of autoimmunity. In MS and EAE, sex-dependent differences in microbial taxa abundance and metabolite production have been associated with differential immune activation and disease severity, although causal relationships remain incompletely defined. Importantly, sex hormones may influence microbial metabolic output, including SCFA production and bile acid signaling, thereby indirectly modulating immune tone and barrier integrity. However, most human MS microbiome studies remain underpowered to resolve sex-specific effects, and few integrate hormonal, microbial, and immune parameters simultaneously. Future studies incorporating sex-stratified analyses and longitudinal hormone–microbiota–immune profiling will be essential to clarify mechanistic links and to inform sex-aware therapeutic strategies.

## Challenges and future directions in MS microbiome research

Despite rapid advances in MS microbiome research, several critical challenges limit mechanistic interpretation and clinical translation. First, methodological heterogeneity across studies including variability in sequencing platforms, analytical pipelines, and functional inference continues to confound cross-cohort and cross-regional comparisons. Harmonization of experimental design, data processing, and reporting standards (e.g., STORMS guidelines) remains a prerequisite for reproducible discovery and meaningful comparison across studies [94]. Second, most current evidence is correlative rather than causal, relying heavily on cross-sectional designs and preclinical models. Establishing causality will require longitudinal human studies combined with functional validation using metabolomics, immune phenotyping, and targeted microbial or metabolite-based interventions [8]. It is therefore useful to grade the mechanistic maturity of the pathways proposed here. Among the links discussed in this review, short-chain fatty acid–mediated regulatory T-cell induction and epithelial/barrier reinforcement carry the strongest support, resting on convergent human association data, gnotobiotic and EAE experiments, and defined molecular mediators (GPR41/43 signalling, FOXP3 induction). Polysaccharide A–driven Treg/IL-10 programming and propionate-related immunometabolic effects are similarly well supported, with both mechanistic and early interventional human data. By contrast, molecular mimicry between microbial and myelin antigens, and sex hormone–microbiota–immune interactions, remain biologically plausible but largely inferential in human MS, and will require direct causal testing — for example, T-cell receptor and peptide-homology mapping, and sex-stratified longitudinal cohorts with hormone tracking.

Limited species-level resolution in many studies further constrains mechanistic inference, particularly when functionally distinct taxa (e.g., *Faecalibacterium duncaniae* versus *F. prausnitzii*) cannot be reliably differentiated using short-read 16 S sequencing approaches. Third, regional and demographic underrepresentation—especially from non-Western populations limit the generalizability of existing findings. Emerging studies from Egypt and other underrepresented regions demonstrate microbial and metabolic signatures that diverge from those reported in Western cohorts, underscoring the influence of diet, environment, and host context on microbiome–immune interactions [56]. These divergences are biologically meaningful, indicating that reference profiles of a “healthy” microbiome and disease-associated dysbiosis signatures cannot be assumed to transfer across populations without explicit validation. At present, however, data from underrepresented regions are frequently constrained by pilot-scale sample sizes and variability in sequencing depth, which limit the precision and poolability of cross-population inferences. Addressing this gap will depend less on isolated single-center efforts than on coordinated, capacity-sensitive collaboration: cooperative research models—potentially including data-sharing consortia—coupled with harmonized analyses conducted through shared, standardized pipelines could provide a practical and equitable pathway toward strengthening the evidence base in these populations. Without broader geographic representation, microbiome-derived biomarkers and therapeutic strategies risk being overly tailored to Western populations.

**Fig. 5:**
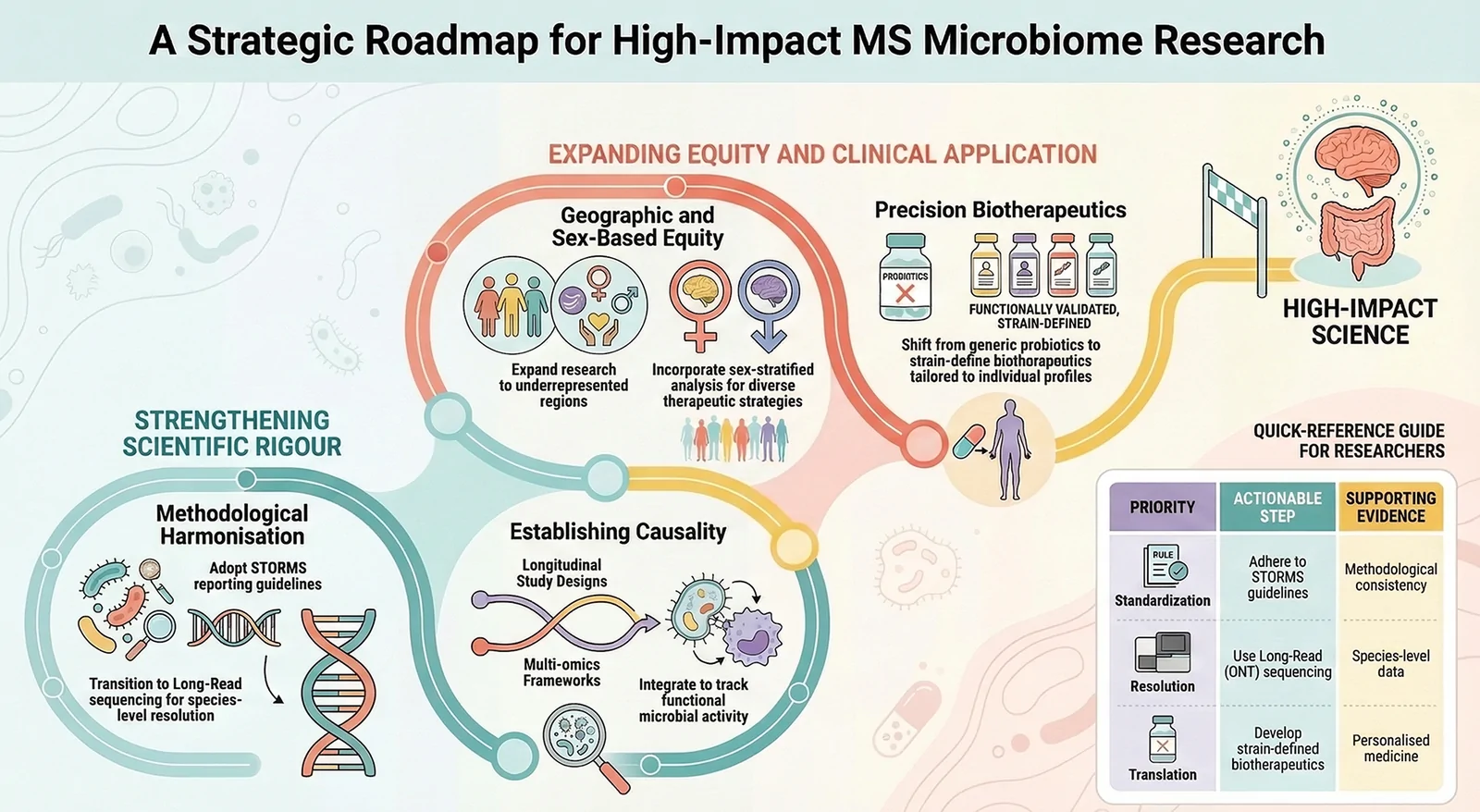
Strategic roadmap for high-impact MS microbiome research. This schematic summarizes the major priorities required to advance the field toward regionally equitable precision medicine, including methodological harmonization, causal and longitudinal multi-omics designs, inclusion of underrepresented populations and sex-stratified analyses, and the development of functionally validated, strain-defined biotherapeutics tailored to individual patient profiles

Finally, translation of microbiota-based interventions will depend on precision stratification that integrates microbial, metabolic, immunological, and host factors, including sex and regional context. Future clinical trials should incorporate mechanistic and functional endpoints such as SCFA levels, immune cell profiling, and longitudinal disability measures to move the field beyond descriptive associations toward predictive and actionable microbiome-informed strategies in MS [95]. These priorities are summarized in Fig. 5, which outlines a strategic roadmap for advancing MS microbiome research toward standardized, causally informed, regionally equitable, and clinically translatable precision medicine.

In conclusion, this review highlights a broadly shared dysbiosis in MS on which distinct regional microbial signatures are superimposed in underrepresented cohorts, underscoring the need for region-specific microbial baselines rather than Western-derived defaults. High-resolution long-read and shotgun metagenomics now provide the species- and strain-level resolution needed to translate these signatures into functional insight. Yet clinical translation of microbiota-targeted strategies — probiotics, SCFA supplementation, and faecal microbiota transplantation — remains constrained by methodological inconsistency, limited causal validation, and the underrepresentation of non-Western populations.

Future research must prioritize standardized multi-omics workflows, diverse and culturally inclusive cohorts, longitudinal study designs, and the development of personalized microbiota-based therapies. As the field evolves from descriptive associations to mechanistic understanding and therapeutic application, integrating global evidence with regional disparities will be essential to fully harness the therapeutic potential of the gut microbiota in MS.

## Data Availability

Not applicable.
